# “It may just be aging”: illness representations of Brazilians with Alzheimer’s disease

**DOI:** 10.1590/0102-311XEN173224

**Published:** 2025-09-01

**Authors:** Paula Gasparini Emery Trindade, Marcia Cristina Nascimento Dourado

**Affiliations:** 1 Universidade Federal do Rio de Janeiro, Rio de Janeiro, Brasil.

**Keywords:** Alzheimer’s Disease, Awareness, Coping Strategies, Dementia, Qualitative Analysis, Doença de Alzheimer, Conscientização, Estratégias de Enfrentamento, Demência, Análise Qualitativa, Enfermedad de Alzheimer, Concienciación, Estrategias de Afrontamiento, Demencia, Evaluación Cualitativa

## Abstract

Different perspectives with regards to illness may be influenced by distinct cultures. The aim of the present study was to explore illness representations of the part of Brazilians with Alzheimer’s disease (AD). A qualitative study was conducted involving 12 participants with mild and moderate AD using a semi-structured interview guide. Interpretative Phenomenological Analysis was used to guide the analysis of the participants’ accounts, resulting in themes that formed five categories. The biological category (six participants) emerged from a group that acknowledged their memory deficits. Two participants from this group mentioned memory deficits and labeled their condition as a disease without the use of diagnostic labels. Three participants from this category recognized memory deficits and related their difficulties to the aging process. The psychosocial category resulted from the accounts of two participants who acknowledged memory deficits and attributed such deficits to the erosion of memory function as well as troubles with day-to-day stress. A mixed category (one participant) included biological, psychosocial, and cultural aspects. The participant labeled the disease as “*Zazá*”, which was considered a cultural euphemism. Two additional categories were identified: one with two participants uncertain of how to make sense of their condition and the last one included one participant who was unaware of her condition. The present results show that illness representations of individuals with AD are influenced by emotional, social, and cultural contexts, and are also deeply embedded in individual coping mechanisms.

## Abstract (pt)

Perspectivas distintas sobre o adoecimento podem resultar de diferentes culturas. Este estudo explora as representações dos brasileiros sobre a doença de Alzheimer (DA), no que diz respeito ao seu adoecimento. É um estudo qualitativo, que foi realizado com 12 participantes (DA leve e moderada) utilizando roteiro de entrevista semiestruturada. A teoria da Análise Fenomenológica Interpretativa orientou a análise das entrevistas. Os relatos dos participantes resultaram em temas, os quais produziram cinco categorias analíticas. A categoria biológica (6 participantes) emergiu do grupo que reconheceu seus déficits de memória. Dois participantes deste grupo mencionaram déficits de memória e rotularam sua condição como uma doença, sem o uso de rótulos diagnósticos. Três participantes reconheceram déficits de memória e relacionaram suas dificuldades ao processo de envelhecimento. A categoria psicossocial resultou de relatos de dois participantes, que reconheceram déficits de memória e os atribuíram a um processo de erosão na função da memória e a problemas com aborrecimentos cotidianos. A categoria mista (1 participante) incluiu aspectos biológicos, psicossociais e culturais. A participante rotulou a doença como “*Zazá*”. Esta expressão foi considerada um eufemismo cultural. Duas categorias adicionais foram identificadas: uma categoria com dois participantes incertos sobre como entender sua condição, e a última incluiu um participante que não demonstrou consciência sobre sua condição. O estudo concluiu que as representações sobre o adoecimento dos participantes com DA são influenciadas por contextos emocionais, sociais e culturais e também estão profundamente enraizadas nos mecanismos de enfrentamento individuais.

## Abstract (es)

Las distintas perspectivas sobre una enfermedad pueden derivar de diferencias culturales. El objetivo de este estudio fue explorar las representaciones que los brasileños tienen de la enfermedad de Alzheimer (EA) con respecto a su enfermedad. Se trata de un estudio cualitativo, realizado con una guía de entrevista semiestructurada y 12 participantes (con EA leve y moderada). La teoría del Análisis Fenomenológico Interpretativo orientó el análisis de las entrevistas. Los relatos de los participantes dieron lugar a temas, que a su vez generaron categorías. El análisis de las entrevistas dio como resultado cinco categorías. La categoría biológica (6 participantes) emergió de un grupo que reconoció sus déficits de memoria. Dos participantes de este grupo mencionaron déficits de memoria y calificaron su condición como una enfermedad, sin utilizar términos diagnósticos. Tres participantes de esta categoría reconocieron los déficits de memoria y relacionaron sus dificultades con el proceso de envejecimiento. La categoría psicosocial resultó de los relatos de dos participantes, que reconocieron los déficits de memoria y los atribuyeron a un proceso de deterioro de la función de la memoria y a problemas con las tareas cotidianas. Una categoría mixta (1 participante) incluyó aspectos biológicos, psicosociales y culturales. El participante denominó la enfermedad como “*Zazá*”, expresión considerada un eufemismo cultural. Se identificaron dos categorías adicionales: una con dos participantes que no sabían cómo interpretar su condición y otra que incluía a una participante que no era consciente de su enfermedad. El estudio concluyó que las representaciones de la enfermedad de los participantes con EA están influenciadas por contextos emocionales, sociales y culturales, y también están profundamente arraigadas en los mecanismos individuales de afrontamiento.

## Introduction

Impaired awareness is a common condition in dementia ^1^. In Alzheimer’s disease (AD), awareness refers to the ability to recognize memory deficits and cognitive impairment caused by the disease process ^2^. This also includes the ability to make judgments and reflections about the causes of impairment ^3^.

Some models ^4^ ^,^ ^5^ have highlighted the role of neuropsychological deficits in impaired awareness phenomena. However, there is a need to explore this issue from a psychosocial standpoint. Such an approach considers social and cultural factors that exert an influence on how individuals live with and manage their condition in everyday life. It is essential to adopt a perspective that prioritizes the voices of those affected by AD ^6^.

A strong threat to self emerges from social interaction in the context of the onset of illness and some individuals may deny their condition ^3^. The way the self is perceived influences elicited responses, which explains why cooperation from others is crucial to the well-being of affected individuals ^3^. However, many cultures stigmatize older people with cognitive impairment, creating a taboo around dementia ^7^. Culture is therefore observed in the perceptions of individuals and their definitions of illness ^7^, which further impacts awareness of the disease. For instance, one study found that black African and Caribbean communities in the United Kingdom viewed dementia as “*a white person’s illness*” ^8^ (p. 1). De Boer et al. ^9^ showed that certain feelings indicate how individuals with AD experience a specific social context. Stigmas related to the condition may disrupt social interactions and cause feelings of inadequacy, embarrassment, humiliation, and isolation, which are commonly expressed in the self-reports of individuals with AD ^9^.

The culture to which individuals belong may determine different perspectives of illness. In Social Psychology, Moscovici devised the Social Representations Theory to understand how knowledge from different fields is appropriated by common sense ^10^. Social Representations Theory refers to a common object; representations are dynamic and shared by groups ^11^ ^,^ ^12^ ^,^ ^13^. This concept encompasses three dimensions: information (knowledge about the object), representation (opinions), and attitude (the way individuals position themselves). Thus, Social Representations Theory involves mutual influences between the social system and individual ^13^.

Hacking ^14^ suggests that certain illnesses have both indifferent and interactive components. The indifferent aspect pertains to the biological causes of illnesses, whereas the interactive component relates to stereotypes associated with individuals suffering from these conditions. From a biomedical standpoint, the stereotype primarily regards a set of symptoms. However, it also has a social dimension, as it is reflected in behavior. Three key elements shape the interaction between classification and those being classified: biological causes, symptoms, and social aspects of the behavior of affected individuals. In this context, the “looping effect” concept describes a cyclical process, when a classification influences an individual’s behavior, which, in turn, alters the stereotype associated with the classification, creating a continuous cycle.

One study investigated the knowledge of 994 Brazilian older adults with regards to AD and their memory loss concerns ^15^. Nearly all participants had heard about AD (95%). However, only 69.5% thought they knew what the condition was ^15^. Moreover, memory problems were considered a part of the normal aging process. The Brazilian population is characterized by moderate to low educational levels. More complete knowledge concerning AD among the participants of the study was only associated with a higher level of education ^15^. These findings suggest that memory loss is poorly addressed in health campaigns on television and in other media channels in the country.

Awareness among individuals living with AD can be influenced by several factors. The capacity of individuals with AD to understand information presented by healthcare providers, updated medical definitions, cultural norms, disease-related stigma, life history, personality, consolidated coping styles, stage of the disease ^16^ ^,^ ^17^ ^,^ ^18^ ^,^ ^19^ ^,^ ^20^ ^,^ ^21^, and diagnostic disclosure may impact an individual’s awareness of the disease.

Two Brazilian surveys were conducted with physicians who were members of national medical societies. The more recent study ^22^ found that most participants (66.7%) usually or always disclose the diagnosis of AD. Compared to the other study conducted 15 years earlier ^23^, this proportion had increased by 22%. An analysis of data from 2008 to 2023 reveals that a barrier related to mental health issues due to the psychological distress of individuals with AD has not yet been overcome, exerting an influence on diagnostic disclosure ^22^. Moreover, the degree of uncertainty with regards to the diagnosis, considering biomarkers, impacts the decision to disclose the diagnosis or not ^22^.

Studies exploring the illness representations of Brazilians with AD are lacking. Cultural aspects, social stigma, and threats to self may impact how individuals express their awareness of the disease. The aim of the present study was to explore specificities in the illness representations of Brazilians with AD. It is particularly relevant to value the self-reports of such individuals regarding their experience of living with the disease.

## Method

### Design

The present qualitative study involved the narratives of individuals with AD in terms of their awareness of the disease and their illness representations.

### Participants

This study included 12 participants with AD (seven women). The participants were recruited from the Center for Alzheimer’s Disease and Related Disorders, Institute of Psychiatry, Federal University of Rio de Janeiro (Brazil). A psychiatrist performed the clinical diagnosis of AD based on imaging studies, laboratory tests, cognitive screening tests, and a clinical interview. All participants were diagnosed with major neurocognitive disorder according to the fifth edition of the *Diagnostic and Statistical Manual of Mental Disorders* (DSM-5) ^24^.

The group consisted of seven individuals with mild AD (CDR1) and five with moderate AD (CDR2) according to the *Clinical Dementia Rating* (CDR) scale ^25^, which is used to assess the severity of dementia. Another inclusion criterion was a score of 12 to 26 on the *Mini-Mental State Examination* ^26^, which is used assess cognitive performance through tasks involving attention, calculation, immediate memory, recall, language, and orientation (spatial and temporal). The exclusion criteria were epilepsy, aphasia, a history of head trauma, and a history of alcohol abuse. All participants were receiving medical treatment at the time of the interview, including cholinesterase inhibitors.

Table 1 displays the clinical and demographic characteristics of the sample.

**Table 1:** Clinical and demographic data of sample.

Participants with AD	Mean	SD
Age	75.00	8.82
Years of education	11.67	3.7
Years since diagnosis of dementia	4.33	2.02
Number of children	3.17	1.64
MMSE score	20.42	2.81
	n	%
CDR 1	7	58
CDR 2	5	42
Marital status		
Married	6	50
Widowed	4	33
Separated or divorced	2	17
Sex		
Female	7	58
Male	5	42
Caregiver		
Yes	12	100
Financial situation		
Retired	8	67
Sick pay	2	17
Dependent on caregiver	2	17

AD: Alzheimer’s disease; CDR: *Clinical Dementia Rating* scale (CDR1 - mild dementia; CDR2 - moderate dementia); MMSE: *Mini-Mental State Examination*; SD: standard deviation.

### Interviews

Interviews were conducted with open- and closed-ended questions based on the *Assessment Scale of Psychosocial Impact of the Diagnosis of Dementia* (ASPIDD) ^2^. The interview guide contained 24 questions in different domains: (1) cognitive deficits and health condition, (2) family and social relationships, (3) emotional status, and (4) functional status (instrumental and basic activities of daily living). Depending on the answers, additional questions were also asked. P.G.E.T. contacted the caregivers of the participants (family and professional caregivers) and invited them to participate in the study. Next, the researcher explained the objective of the study and the required participant assessments. Interviews were conducted and transcribed in Brazilian Portuguese and then translated into English by P.G.E.T. The interviews were recorded and compiled on an Excel spreadsheet (https://products.office.com/) to preserve the participants’ own words.

Dementia requires more flexible analytical approaches, as individuals suffering from the illness may have communications problems. Therefore, the *Results* section includes answers related to all awareness domains to provide more details on the main topic. One question specifically explored illness representations.

Box 1 displays the questions in the interview guide.

**Box 1:** Interview guide.

#	QUESTION
1	Can you identify anything wrong with you? (If necessary) What?
1.1	Do you think you have a health condition or problem?
2	(If necessary) What about memory lapses? Do you have them?
3	Has anyone ever talked to you about this problem?
4	What do you perceive about your condition?
5	Are you sadder now than before? (If yes) Why?
6	Are you more anxious than before? (If yes) Why?
7	Are you angrier than before? (If yes) Why?
8	Do you have friends?
9	Did you used to visit people? (If not) why?
10	Did you usually receive visits at home? (If not) why?
11	Has your desire to be with other people changed? (If yes) why?
12	How do you think people feel about you these days?
13	And how do you feel about people?
14	How is your relationship with your family?
15	Can you identify any change in the way your family treats you? (If necessary) What changes?
16	How do you feel about the care you receive?
17	How is your relationship with your caregiver?
18	What difficulties do you have these days?
19	Has your routine changed?
19.1	(If necessary) How?
20	How do you think health problems influence your life?
20.1	How do you feel about this?
21	Do you ask for or need help to do tasks? (If necessary) What tasks? Help from whom?

### Analyses

The theory of Interpretative Phenomenological Analysis (IPA) was selected for the analysis of the interviews ^27^. IPA is an approach to explore how individuals make sense of their experiences and is an effective method used to access the singular meanings (thoughts and feelings) of the interviewee ^27^. IPA is useful for exploring, describing, contextualizing, and interpreting perceptions ^28^. It involves an active individual process of interpreting experiences and everything in the inner universe of individuals (objects, other individuals, and events). In this process, individuals seek to assign meanings that emerge from their own experiences ^29^. The theory also addresses the role of interactions in the social world. Therefore, beyond idiosyncrasies, the interpretations of individuals are influenced by shared social processes, resulting in a symbolic interactionist perspective ^29^.

P.G.E.T. and M.C.N.D. read all the interviews independently twice. In the interviews, P.G.E.T. used keywords, which were analyzed together by the researchers, with the aim of identifying themes and connections between themes ^27^. P.G.E.T. then produced a table with themes concerning the illness representations of the participants ^27^. To comply with reliability and transparency criteria, the researchers detailed each aspect of the data collection process, explored excerpts from the textual data, and described the criteria used to analyze the data ^30^.

### Ethics

The authors followed the ethical guidelines outlined in the revised *Declaration of Helsinki*. This study received approval from the Institutional Review Board of the Institute of Psychiatry, Federal University of Rio de Janeiro (certificate n. 92966318.3.0000.5263). All participants and caregivers received oral and written clarifications regarding the study and provided written consent prior to the interview.

## Results

Five themes and some subthemes emerged from the interviews (Box 2).

The results were arranged in dialogues between the interviewer and participant. For the purposes of the present study, the simple perception of memory deficits was considered awareness of the disease.

**Box 2:** Thematic analysis of interviews.

MAIN THEMES			
Biological category: Disease Aging Nervous system	Psychosocial category: Erosion process Irritation	Mixed category (biological, psychosocial, and cultural aspects)	“Don’t know how to explain” category	Illness unawareness category

### Biological category

Participants who labeled the condition as a disease and those who attributed memory deficits to the nervous system or an aging process were grouped into this category. The biological category captured how the participants understand and make sense of their memory deficits through a biomedical or physiological lens, often drawing upon broader cultural narratives of disease, aging, or bodily systems. Although the participants employed biological explanations, their narratives were deeply intertwined with emotional and existential meanings that go beyond clinical labeling.

#### Disease

Participants who explicitly referred to their condition as a “disease” exhibited a heightened awareness of their cognitive decline. However, this awareness did not equate to understanding, as it was accompanied by uncertainty, distress, and emotional vulnerability.

One participant was aware of her illness. Her identification of having “*a mental disease that has no cure*” reflects more than factual knowledge; it suggests a confrontation with a new, uncertain identity. Her expressions of disconnection and desire to “*disappear*” indicate existential distress and social withdrawal. Her sense-making involved both a clinical interpretation and a deeply personal struggle with meaning and selfhood.

“(Memory deficits?) *Forgetfulness. I did some imaging tests and the forgetfulness was getting worse.* (Sadder?) *Some days are worse, others better, wishing to disappear.* (What do you perceive about your condition?) *I have a mental disease that has no cure.* (Has the desire to be with other people changed?) *Yes, I have become disconnected from everything. The world could come to an end and I would still be disconnected.* (How do health problems influence your life?) *I can’t leave the house without taking a list, because I forget things...*” (C, female, moderate AD, 68 years old).

One participant revealed her awareness the disease by mentioning vulnerability and emotional changes resulting from her condition. Initially, she did not know how to explain her condition; however, when additional questions were asked, she mentioned the word “*disease*”. Therefore, the acknowledgment of her condition as a disease was not through direct understanding, but rather through a process of inquiry and reflection triggered by the interview. Her hesitation (“*I don’t know*”) gradually gave way to the recognition that forgetting “*on purpose*” was impossible, thus interpreting her experience as pathological.

“(Memory deficits?) *Sometimes my memory fails, and when I talk, I repeat things.* (Has anyone ever talked to you about your memory deficits?) *I don’t know.* (Would you like to know?) *Yes, to know what is happening to me.* (What difference would it make?) *I think it’s a disease because it’s impossible to forget on purpose.* (More anxious?) *I get embarrassed because I repeat the same word two or three times.* (What do you perceive about your condition?) *It just happened.* (How do people feel about you these days?) *That I am senile, sometimes they look suspicious, but it’s not happening that much nowadays.* (And how do you feel about other people?) *Ashamed, shy*” (M, female, moderate AD, 65 years old).

In both cases, the label “*disease*” serves as a framework through which the participants interpret their cognitive loss, yet their reflections reveal a complex process of meaning-making that encompasses emotional suffering, identity shifts, and interpersonal fears.

#### Aging

Participants in this subtheme constructed their memory issues within the broader context of aging, which is a socially normalized, less stigmatizing framework than “*disease*”. This may serve as a coping mechanism, enabling the maintenance of a sense of continuity and normalcy despite cognitive changes.

One participant was considered aware of his disease. He attributed his condition to aging, indicating a possibly protective narrative that normalizes cognitive decline by identifying memory loss as a “*natural process*”, thus minimizing its impact on daily life:

“(Memory deficits?) *Yes, I am aware of that. I forget more easily. I even joke, ‘It’s not that I forget, it’s just that I don’t remember’. But it’s a natural process, isn’t it?* (What do you perceive about your condition?) *It’s about aging.* (Do you currently have any difficulties?) *No, nothing that affects me at all*” (G, male, mild AD, 82 years old).

One participant was aware of his disease and stated that aging was the reason for his memory deficits:

“(Memory deficits?) *I don’t know, because of a mental block, forgetfulness.* (Has anyone ever talked to you about this problem?) *No, I would like to know, to understand what it’s all about.* (What do you perceive about your condition?) *It may just be aging.* (Changes in the way your family treats you?) *No, they are very caring with me*” (A, male, mild AD, 66 years old).

Another participant was also considered aware of the disease. She attributed her condition to the passage of time and aging, and emphasized specific difficulties: 

“(Memory deficits?) *I forget everything. My mind fades.* (Has anyone ever talked to you about your memory deficits?) *No.* (Would you like to know?) *Yes, I would, but the problem is that my mind is damaged.* (What do you perceive about your condition?) *I don’t know what the explanation is. It’s about the passing of time. I’m not always like this. Sometimes, depending on the subject discussed, my situation gets better*” (H, female, moderate AD, 80 years old).

Although she identifies fluctuations in her cognition (“*sometimes... my situation gets better*”), there is a palpable uncertainty in her meaning-making, revealing both awareness and disorientation.

#### Nervous system

One participant acknowledged his memory deficits, despite his attempts to hide such deficits from others. The interplay between tension, nervousness, and memory loss points to an embodied understanding of his condition. While he acknowledges forgetfulness, his decision to conceal it from others suggests stigma or fear of social judgment:

“(What do you perceive about your condition?) *It’s about the nervous system. Tension and nervousness dominate my nervous system, making me forget.* (How do you feel about people these days?) *Forgetfulness is my problem, so I don’t mention it.* (Changes in the way your family treats you?) *No, forgetfulness does not affect me*” (W, male, mild AD, 76 years old).

### Psychosocial category

Psychosocial causes mentioned by the participants composed this category, which encompasses efforts on the part of the participants to make sense of their memory impairments by attributing them to life circumstances, emotional states, or accumulated stress rather than strictly biological causes. This category reveals a more nuanced, layered understanding of the illness by which meaning is constructed through lived experiences and situated within broader life narratives.

#### Erosion process

One participant tended to downplay his memory deficits and was therefore considered partially aware of the disease:

“(Memory deficits?) *No, I think I am doing very well for my age. I am not very forgetful.* (What kind of forgetfulness do you have?) *Well, I forgot.* (Is it a word that does not come to mind spontaneously or an object that you misplaced?) *No, I don’t think so.* (What do you perceive about your condition?) *I think it may be a kind of cluttering.* (Did you used to study a lot, used to work a lot?) *Oh, I worked my entire life.* (Do you think your memory has kind of eroded!?) *Yes, a little bit.* (What difficulties do you have these days?) *No serious difficulties. I don’t have any problems*” (D, male, mild AD, 89 years old).

The use of the word “*cluttering*” and agreement with the interviewer’s term “*eroded*” suggests an internalization of the idea that his cognitive difficulties are the result of a long-term accumulation of life experiences, such as hard work and aging.

#### Hassles

One participant recognized her memory deficits:

“(Memory deficits?) *I got sadder, melancholic, and lonelier. Sometimes my memory plays tricks on me and keeps me talking.* (Has anyone ever talked to you about your memory deficits?) *No, people sometimes say it’s a lack (of attention.* (What do you perceive about your condition?) *I don’t know if it’s because of hassles.* (How do you feel about people?) *Not bad, I tell people that I forget*” (M, female, mild AD, 65 years old).

She acknowledges sadness, loneliness, and melancholy, suggesting a view of cognitive decline intertwined with emotional and relational challenges. Her memory “*playing tricks*” reflects ambivalence towards the condition - it is recognized, but also mysterious and elusive.

### Mixed category (biological/psychosocial/cultural aspects)

The mixed category included only one participant, who was considered aware of the disease. She expressed that a disease was the cause of her condition and mentioned a combined explanation, ranging from the aging process to emotional distress:

“(Memory deficits?) *Listen, I won’t say that I’m very senile, but a little senile.* (What do you perceive about your condition?) *It’s about ‘Zazá’* [AD]*. It’s tragic when Zazá hits you like that!* (Sadder?) *The weird thing is that I’m not* [laughs]*. Because if I think, ‘I’m going crazy’, it’ll be worse, then I’ll really go crazy. So, I need to go on and work with what I can.* (Angrier?) *Sometimes.* (But is it related to memory?) *I don’t know, it may be related and I’m not aware of it.* (Changes in routine) *No, because, for example, when there’s something that I need to do, I write it down and keep it there. For example, here...* [searching for a word] *Hey, guys, this is irritating me, because I don’t know why this is happening. I can’t speak properly, honestly, I can’t tell you what it is that makes me like this from time to time, but I do things at home, my family does the shopping, and I write down everything I want. I buy things and throw the list away*” (S, female, mild AD, 79 years old).

This category illustrates how the meaning-making process around AD is multi-layered, involving biological, emotional, and culturally shaped understandings. Her narrative reveals an attempt to preserve autonomy and optimism even in the face of acknowledged decline, and reflects a dynamic negotiation of self-identity, by which she integrates biological awareness, emotional resilience, and cultural narratives to construct a livable, empowered understanding of AD.

### Category “don’t know how to explain”

This category captures ambivalence and partial insight, where the participants struggle to articulate the nature of their condition. Their sense-making is shaped by emotional, cognitive, and relational dynamics, and often marked by indirect awareness. One participant expressed partially impaired awareness of the disease; she failed to answer the question on her condition properly:

“(Memory deficits?) *Listen, I don’t think so. Sometimes I have a problem with my back and I suffer from hypertension, but I treat myself, I take medication. So far, I don’t feel sick.* (What about forgetfulness?) *Sometimes I forget little things, but it’s not the kind of forgetfulness that erases.* (Give me an example) *If I have a lot of tasks to do, my mind slows down. I get more worried if I have a lot of things to do. For example, I study theology and when I run into difficulties, I withdraw from a lot of things I want to do. I don’t give up that easily. I like to insist, even though I have some difficulty...* (What do you perceive about your condition?) *Listen, I don’t forget a lot or erase things. I think it’s normal. I don’t think that a single person never forgets anything*” (E, female, mild AD, 80 years old).

Another participant did not know how to explain his condition. However, he stated that he had delegated decisions about his own life to his wife. He was thus considered aware of the disease:

“(Memory deficits?) *I like being with my wife because I think I’m fine, you know? But I know that I have been forgetful.* (What do you perceive about your condition?) *I think my wife knows, do you understand?* (But what do you think it is about?) *Honestly, I don’t know how to explain it. It may be about lapses. But I am not that forgetful.* (Angrier?) *I would prefer to have my wife answer for me. As long as I’m close to her, I don’t feel anxiety. If she says I should not go somewhere, I don’t go. I am very conscious of this*” (M, male, moderate AD, 72 years old).

### Illness unawareness category

One participant promptly denied her condition. She was considered unaware of the disease, as she took her condition for granted, explaining it as part of the aging process.

“(Memory deficits?) *Not at the present moment, because I have always taken care of myself, didn’t drink, didn’t smoke. I just studied and worked. Now I’m here. I’ve forgotten the past and the future belongs to God.* (Have you not been forgetful?) *I don’t know. If I forget, I have already forgotten.* (What do you perceive about your condition?) *I am well cared for, thank God, that is what we study for, right? But due to the nature of life, we forget things, right?*” (L, female, moderate AD, 82 years old).

This narrative functions as a meaning-making structure that preserves dignity, order, and continuity of identity. The refusal to accept forgetfulness as a disease protects her from the social and psychological disruption that a diagnosis may bring.

## Discussion

The aim of this study was to investigate how individuals with mild and moderate AD represent their illness. One group (one participant) acknowledged the disease using diagnostic labels. The second group (two participants) mentioned disease and memory deficits, without diagnostic labels. The third group (three participants) recognized their difficulties as part of the aging process. In line with Clare et al. ^31^, the fourth group (three participants) was uncertain of how to make sense of their condition and the fifth group (one participant) did not recognize any difficulties. We also found a sixth group (two participants) that recognized memory deficits but did not mention a disease.

Our findings partially align with those of a previous study by our research group, which also found both singular and mixed explanatory models combining biological/genetic and psychosocial dimensions ^32^. Both studies also identified a category that included participants who did not know how to explain their condition, underscoring the centrality of ambiguity in AD representation.

Concerning the biological category, there was a key distinction between participants who recognized their condition as pathological and used diagnostic labels from others who only recognized memory deficits but attributed them to aging. In the biological category, the participants drew upon dominant biomedical and aging narratives to make sense of their experiences. However, their interpretations were far from passive reflections of medical knowledge. Instead, they actively constructed meanings that interwove emotional, relational, and existential dimensions. Whether through the language of disease, aging, or nervous system dysfunction, each participant’s account reveals unique coping strategies, degrees of acceptance, and efforts to maintain identity in the face of cognitive decline. As a previous study pointed out, while age is a risk factor for dementia, it is not synonymous with illness, despite persistent assumptions in certain cultures ^33^.

In the psychosocial category, the participants framed memory loss not through medical labels, but through the lens of life history, emotional burden, and everyday stressors. These narratives reflect complex processes of meaning-making in which cognitive symptoms are embedded in the broader landscape of lived experience. While biological explanations can impose a definitive identity (e.g., “patient”, “diseased”), psychosocial interpretations enable more fluid, personally resonant understandings that protect self-esteem, the continuity of identity, and social belonging.

The mixed category (represented by a single participant) offered a compelling illustration of fluid, layered meaning-making. This individual combined biological and psychosocial explanations with a culturally resonant euphemism - referring to AD as “*Zazá*”. This naming reflects both personalization and downplaying of the disease, which are consistent with cultural norms favoring humor, affective bonds, and lightness in the face of adversity. The participant’s narrative blended emotional resilience with practical adaptations, such as using notes, to maintain a sense of autonomy.

In the “don’t know how to explain” category, the participants hovered in a space between awareness and avoidance, with some shifting their sense-making onto relational figures or stress-based explanations. In the “illness unawareness” category, meaning was preserved through life narratives of health, morality, and divine trust, enabling individuals to distance themselves from the disruptive implications of the disease. The different categories show that diagnosis alone does not determine how individuals understand the experience.

From a medical anthropological standpoint, these diverse representations highlight the culturally embedded nature of the illness. According to Geertz ^34^, culture is the context in which different events become intelligible. The author highlights the importance of culture in the construction of every human phenomenon ^35^. From this perspective, perceptions, interpretations, and actions are culturally constructed, even in the health field ^35^. Eisenberg ^36^ distinguished the “illness process”, which refers to abnormalities in the structure or functioning of organs or systems, from the “illness experience”, which refers to the subjective experience of patient discomfort ^35^. Therefore, the experience of illness does not refer to a simple reflection on the pathological process (biomedical sense). It combines individual and collective norms, values, and expectations, and is expressed in specific ways of thinking and acting ^35^.

While it is not possible to quantify the exact contribution of biological, psychosocial, and cultural factors to illness awareness, it is important to acknowledge that AD is marked by cognitive decline, which may itself impair the ability to recognize symptoms. This may explain the contradictions in the discourse of the participants - such as recognizing symptoms without acknowledging illness. The concept of “looping effect” ^14^ is useful here. For example, AD-specific information is considered to be negative and can have an adverse impact upon those receiving the information. Therefore, diagnostic labels can alter behavior and identity, sometimes leading to denial or resistance due to stigma or fear of the loss of autonomy ^37^.

On the other hand, acknowledging the disease may be related to an integrative attitude, which occurs when an individual incorporates changes to the self ^38^. The three participants who mentioned “*disease*” reported coping strategies to deal with its consequences. One stated that she tells family members what she is doing so that they can monitor her. The other two reported using lists to help remember things in their daily lives. Interestingly, two of these participants did not express concerns over the development of their condition. The lack of reflection on their future may be an effective strategy to cope with the illness by maintaining selfhood despite cognitive loss.

Other coping strategies involved downplaying memory deficits, attributing changes to aging, a mental block, an eroded memory, a natural process, or a lack of attention, not reflecting on the condition and the delegation of personal care to a family member. The almost amused way in which one participant labeled her condition “*Zazá*” was considered a cultural downplaying of the illness. Such coping strategies reflect the theme “*I want to be me*” identified in one study ^39^ and the self-protection category ^38^, reflecting the intention to maintain a sense of self and identity. The theme “*It will get worse*” ^39^ was identified in only one participant in our study, who mentioned a “*disease with no cure*”.

Cultural dimensions were also salient. In Latin American culture, adult daughters and sons are responsible for the care of older people ^40^. In Brazil, family members, especially wives and daughters, are usually responsible for caring for individuals with dementia ^41^. Moving to long-term care facilities is uncommon, which may delay awareness transitions that, in other contexts (e.g., Norway), are triggered by institutionalization ^41^. Religious beliefs may also come into play. In a Brazilian qualitative study involving individuals with mild and moderate AD, only one participant mentioned supernatural causes for the disease ^32^. No participants mentioned supernatural causes in the present study, suggesting that Brazilians may tend to adopt a natural perspective towards AD.

Further studies on diagnostic disclosure are needed in Brazil, as receiving a diagnostic label may impact the illness representations of affected individuals.

### Strengths and limitations

This study offers a nuanced understanding of how Brazilians with mild and moderate AD interpret and make sense of their experiences with the condition. Through the lens of IPA, the findings underscore the subjective and contextual dimensions of illness representations, highlighting the role of biological, psychosocial, and cultural influences in shaping awareness and coping strategies. However, using an interview guide based on a preexisting scale may have biased the findings due to the more directive feature of this kind of interview, potentially disrupting the development of other aspects of the participants’ answers. Moreover, the type of recruitment may have biased the findings, as all participants were receiving treatment at a reference center for AD.

## Conclusion

Illness representations of the participants with AD are deeply embedded in individual coping mechanisms and influenced by social, emotional, and cultural contexts. These representations underscore the importance of recognizing diverse forms of awareness and the protection strategies individuals use to preserve their identity and autonomy in the face of cognitive decline. The findings reveal that understanding and supporting individuals with AD requires more than simply conveying medical information; it requires a person-centered approach that respects the ways by which individuals construct meaning around their condition. To improve care outcomes, healthcare providers should develop more culturally responsive, flexible, empathetic approaches that accommodate the subjective experiences of individuals with AD. Greater attention to how diagnoses are disclosed and how individuals and families interpret diagnoses is crucial to promoting effective support systems and reducing stigma.

## References

[1] Fischer A, Dourado MCN, Laks J, Landeira-Fernandez J, Morris RG, Mograbi DC (2019). Modelling the impact of functionality, cognition, and mood state on awareness in people with Alzheimer's disease. Int Psychogeriatr.

[2] Dourado MC, Mograbi DC, Santos RL, Souza MF, Nogueira ML, Belfort T (2014). Awareness of disease in dementia factor structure of the assessment scale of psychosocial impact of the diagnosis of dementia. J Alzheimers Dis.

[3] Clare L (2003). Managing threats to self awareness in early-stage Alzheimer's disease. Soc Sci Med.

[4] Morris RG, Morris RG, Becker JT (2004). The cognitive neuropsychology of Alzheimer's disease.

[5] Rosen HJ (2011). Anosognosia in neurodegenerative disease. Neurocase.

[6] Romaioli D, Pesce E, Chiara G (2025). Social representations of dementia. A qualitative inquiry into perspectives of people with dementia, professionals, and informal caregivers.. Dementia (London).

[7] Cipriani G, Borin G (2015). Understanding dementia in the sociocultural context a review. Int J Soc Psychiatry.

[8] Berwald S, Roche M, Adelman S, Mukadam N, Livingston G (2016). Black African and Caribbean British communities' perceptions of memory problems "we don't do dementia". PLoS. One.

[9] de Boer ME, Hertogh CM, Dröes RM, Riphagen II, Jonker C, Eefsting JA (2007). Suffering from dementia - the patient's perspective a review of the literature. Int Psychogeriatr.

[10] Gomes R, Mendonça EA, Pontes ML (2002). Social representations and the experience of illness. Cad Saúde Pública.

[11] Sancovschi B (2007). On the notion of representation in S Moscovici and F. Varela. Psicol Soc.

[12] Coelho MTAD, Carvalho VP, Porcino C (2019). Social representations of disease, uses and meanings attributed to integrative and complementary practices by students. Saúde Debate.

[13] Moscovici S (2012). A psicanálise, sua imagem e seu público.

[14] Hacking I (2007). Kinds of people moving targets. Proc Br Acad.

[15] Matioli MNPS, Etzel A, Prats JAGG, Medeiros WFO, Monteiro TR, Soares AM (2011). Worries about memory loss and knowledge on Alzheimer's disease in community-dwelling elderly from Brazil. Dement Neuropsychol.

[16] Clare L, Pratt R (2005). Perceptions of change over time in early-stage Alzheimer's disease. Dementia.

[17] Clare L, Wilson BA (2006). Longitudinal assessment of awareness in early-stage Alzheimer's disease using comparable questionnaire-based and performance-based measures a prospective one-year follow-up study. Aging Ment Health.

[18] Johannessen A, Engedal K, Haugen PK, Dourado MCN, Thorsen K (2018). "To be, or not to be": experiencing deterioration among people with young onset dementia living alone.. Int J Qual Stud Health Well-being.

[19] Thorsen K, Dourado MCN, Johannessen A (2020). Awareness of dementia and coping to preserve quality of life a five-year longitudinal narrative study. Int J Qual Stud Health Well-being.

[20] Rose L (2003). Larry's way: another look at Alzheimer's from the inside..

[21] Voris E, Shabahangi N, Fox P (2009). Conversation with Ed: eaiting for forgetfulness. Why are we so afraid of Alzheimer's disease? s.l.:. Elders Academy Press.

[22] Souza VS, Guazzelli SB, Cruz LC, Resende EPF, Souza LC, Barbosa MT (2023). Diagnostic disclosure of Alzheimer's disease in Brazil a national survey of specialized physicians. Arq Neuropsiquiatr.

[23] Raicher I, Shimizu MM, Takahashi DY, Nitrini R, Caramelli P (2008). Alzheimer's disease diagnosis disclosure in Brazil a survey of specialized physicians' current practice and attitudes. Int Psychogeriatr.

[24] American Psychiatric Association (2013). Diagnostic and statistical manual of mental disorders, fifth edition (DSM-V).

[25] Maia AL, Godinho C, Ferreira ED, Almeida V, Schuh A, Kaye J (2006). Application of the Brazilian version of the CDR scale in samples of dementia patients. Arq Neuropsiquiatr.

[26] Bertolucci PH, Brucki SM, Campacci SR, Juliano Y (1994). O mini-exame do estado mental em uma população geral impacto da escolaridade. Arq Neuropsiquiatr.

[27] Smith JA, Osborn M, Jarman M, Murray M, Chamberlain K (1999). Qualitative health psychology: theories and methods.

[28] Tombolato MA, Santos MA (2020). Interpretative Phenomenological Analysis (IPA) basic underpinnings and applications in research. Rev Abordagem Gestál.

[29] Freire MRL, Nascimento AM, Roazzi A (2021). Análise Fenomenológica Interpretativa (AFI) enlaces com a psicologia cognitiva. Revista Amazônica.

[30] Tuval-Mashiach R (2017). Raising the curtain the importance of transparency in qualitative research. Qualitative Psychology.

[31] Clare L, Gamble LD, Matyr A, Quinn C, Litherland R, Morris RG (2022). Psychological processes in adapting to dementia illness representations among the IDEAL cohort. Psychol Aging.

[32] Trindade PGE, Santos RL, Lacerda I, Johannessen A, Dourado MNC (2019). Awareness of disease in Alzheimer's disease what do patients realize about their own condition?. Aging Ment Health.

[33] Lima AMM, Silva HS, Galhardoni R (2008). Successful aging paths for a construct and new frontiers. Interface (Botucatu).

[34] Geertz C (1973). The interpretation of cultures.

[35] Uchôa E, Vidal JM (1994). Antropologia médica elementos conceituais e metodológicos para uma abordagem da saúde e da doença. Cad Saúde Pública.

[36] Eisenberg L (1977). Disease and illness Distinctions between profesiional and popular ideas of sickness. Cult Med Psychiatry.

[37] Jerzy FSB, Brzozowski A, Caponi S (2010). Interactive classifications the case of attention-deficit hyperactivity disorder in children. Interface (Botucatu).

[38] Clare L (2002). We'll fight it as long as we can coping with the onset of Alzheimer's disease. Aging Ment Health.

[39] Harman G, Clare L (2006). Illness representations and lived experience in early-stage dementia. Qual Health Res.

[40] Santos RL, de Sousa MFB, Ganem AC, Silva TV, Dourado MCN (2013). Cultural aspects in dementia differences in the awareness of Brazilian caregivers. Trends Psychiatry Psychother.

[41] Oliveira AP, Caldana RHL (2012). As repercussões do cuidado na vida do cuidador familiar do idoso com doença de Alzheimer. Saúde Soc.

